# Strategic use of observer-perspective questions in couples therapy

**DOI:** 10.3389/fpsyg.2023.1229991

**Published:** 2023-08-31

**Authors:** Peter Muntigl, Adam O. Horvath

**Affiliations:** ^1^Faculty of Education, Simon Fraser University, Burnaby, BC, Canada; ^2^Department of Translation, Interpreting and Communication, Ghent University, Ghent, Belgium

**Keywords:** conversation analysis, epistemics, questions, reflexive questions, relationship, couples therapy, emotions

## Abstract

Questions are one of the most frequently used strategies in therapy. There is a body of theoretical work on the kinds of questions that are preferred in specific treatment approaches. However, research on the use of questions in general, how they are formed and what specific therapeutic work they do, is relatively scarce in the literature. In this study, we use the conceptual framework and methods of conversation analysis (CA) to examine how systemic questions soliciting clients' perspective on the partners' thoughts and intents (Observer-Perspective Questions; OPQs) are realized interactively in actual clinical practice and the range of therapeutic work they perform in couples therapy. We identified 78 OPQs from archival data of videotaped time-limited couples therapies, a clinical population working with a professional therapist. From this set of 78 OPQs, five excerpts representing diverse use of OPQs were selected. These excerpts were transcribed in detail capturing not only the textual content but also the prosodic, gestural, and non-verbal aspects of these episodes. Using CA methodology, we identified four specific kinds of changes these questions can promote: progress toward relational optimism, support of positive aspects of the couple's relationship, promoting the concept that the couples' experiences and emotions are interlinked, and introducing new creative relational options. Detailed CA analyses of these clinical excerpts allowed us to identify how the OPQ sequences were built to realize these therapeutically useful moves using various conversational resources progressively and interactively. The conversational analysis of these sequences facilitated the exploration of relationships between the ways the questions are formed, timed, and delivered and the specific functions they perform to move the therapy forward. In conclusion, we make the general argument that examining important therapy events through a CA perspective provides a significant complementary vector to quantitative research on the therapy process.

## 1. Introduction

Dictionary definitions of “questions” often prioritize notions such as “interrogative sentences or clauses… which are designed to elicit information, defined by syntax or grammatical structure” (e.g., Merriam-Webster, 2019). In the praxis of therapy, the grammatical question form is one of the most frequently used (Hill, 2020) and can be designed to achieve a vast variety of functions; however, eliciting information is a very small subset of the potential designs.

Although therapists commonly ask questions during therapy, the literature (theoretical and empirical) on questions is relatively sparse (Williams, 2023). In most of the available psychotherapy literature, questions are simply divided into two categories: “Open questions”—often using *wh*-*pronoun* forms (e.g., what, who, when, and why); these types of questions are designed to have the effect of encouraging exploration or elaboration on the topic. The second category is “closed questions”; these solicit relatively succinct concrete information or a yes/no answer. Some theories of therapy—e.g., client-centered and humanistic—avoid closed, interrogative questions and favor open-ended formulations (if questions are to be used at all), whereas others (e.g., CBT) are more welcoming to the use of questions and do not promote one type over the other (Elliott et al., 1987; Hill, 2020). Moreover, “open” and “closed” are very course distinctions. Questions in psychotherapy fulfill a diverse and rich range of functions and are a fundamental component of psychotherapy practice.^1^ While questions are ubiquitous in all forms of “talk therapy', some treatment orientations focus on particular types of questions to accomplish specific goals, and the use of these questions is a core element of treatment. For example, *Socratic Questions* are used in Rational Emotive Therapy (RET) and some implementations of CBT, to clarify false and/or dysfunctional beliefs (Ellis, 2019; Beck, 2021); Brief Solution Focused (BSF) therapy uses *Hypothetical* (a.k.a., “Miracle”) *questions* to direct the clients” attention to specific, attainable, but previously neglected solutions (de Shazer et al., 2007). The “Milan School” of Systemic Family Therapy (SFT) developed the notion of *Circular Questions*—soliciting from each participant information about her/his notion of how *other members of the system* think about or perceive certain relational issues. For the Milan version of SFT, these circular questions became a way of re-formulating the identified patient's issue as embedded in a dynamic family context which, they argue, is essential in achieving a collaborative/interactive solution (Palazzoli Selvini et al., 1986).

The most in-depth and influential theoretical work on the use of questions—with a particular focus on family and couples therapy—was offered in a three-part article by Tomm (1987a,b, 1988). Tomm examined the formal properties of questions in the context of therapy and focused on a type of question that is particularly helpful: reflexive question(s) (RQ). He defined RQs as “*Questions asked with the intent to facilitate self-healing in an individual or family by activating the reflexivity among meanings within pre-existing belief systems that enable family members to generate or generalize constructive patterns of cognition and behavior on their own”* (Tomm, 1987b, p. 197). Within this broad category of reflexive questions, he listed eight specific types: Future-oriented questions, Observer-Perspective Questions, [Unexpected] Context-change Questions; Embodied-Suggestion Questions, Normative-Comparison Questions, Distinction-Clarifying questions, Questions Introducing Hypothesis, and Process-Interruption Questions. Tomm's original lists of question types were subsequently critiqued suggesting that, rather than specifying distinct types or forms of questions, there was often considerable overlap between categories and they instead identified the therapeutic work or goals that they were designed to achieve. Nonetheless, these seminal articles continue to enrich and influence our understanding of the range of work questions can do, not only in family and couples therapy but also in individual treatment as well (Collins and Tomm, 2009).

It is important to note, however, that almost all of the current research on this topic^2^ considers the typology and the use of questions in therapy from a theoretical and therapist-oriented perspective. The available literature focuses on the rationale for using questions, identifies several goals to be achieved, and, in many cases, provides hypothetical examples of the use of questions in therapy. Moreover, investigations have tended not to focus on the following important issues: How are questions designed and realized in real clinical contexts? How do they unfold sequentially and interactively? How do questions perform relational work over time when used with couples or families? In short, there is a lack of examination of the kinds of immediate and short-term impacts these questions have on the subsequent discourse. There are studies of the interactional use of questions in everyday conversations (Stivers, 2010), but very few of these studies address the use of questions in the context of the formal discipline of psychotherapy, and, as we saw above, the context of therapy affords special opportunities (and perhaps risks) in the use of questions. The study we present here aims to be a step toward addressing this lacune.

The theoretical and practical bases of our study are grounded in conversation analysis (CA) (Heritage, 2005; Sidnell, 2013). While we are motivated by our overarching goal as stated above, realistically we had to consider the limits of our resources: Within the practice of psychotherapy, questions have a wide range of uses. We therefore selected a relatively narrow (rather than representative) variety of questions for this study: We focus on the category of questions identified by Tomm as “Observer-Perspective Questions” (OPQs) within the context of Narrative treatment modality in which the use of these kinds of questions is closely linked to the theory and method of treatment (White and Epston, 1990; de Shazer et al., 2007).

At a more general level, our research is situated in the context of complementing research approaches to better understand the psychotherapy change process. A majority of research on the psychotherapy process, both theoretical and empirical, focuses on identifying particular interventions and sequences that will result in effective and efficacious treatments of clients' psychological distress. The common theme among these important contributions is an attempt to answer the “*what”* [works] question of the therapy. The important “*how*” [it's made to work] aspect of the change process is, by and large, left in the margins in these designs. CA-based research takes a complementary perspective and focuses on how important events in therapy unfold in “talk therapy”; how these interventions are realized and made effective. For the most part, the focus of these CA investigations in CT and FT has been on the therapeutic alliance (Muntigl and Horvath, 2016), therapist-client collaboration (Sutherland and Strong, 2011), resistance (Muntigl, 2013), so-called “change moments” (Couture, 2006, 2007), therapeutic agendas (Gale, 1991), spouses claiming independence and control (Janusz et al., 2021), ascriptions of blame (Buttny, 1993; Edwards, 1995), client complaints (O'Reilly, 2005; Peräkylä et al., 2023), and the familial moral order (Hutchby and O'Reilly, 2010; Wahlström, 2016). A “critical methodological review” arguing for the benefits of using CA to study family therapy is given by Tseliou (2013). We hope that the research we present in this study will not only serve to better understand how OPQs work in couples therapy but also strengthen the argument that CA research into the psychotherapy process can provide an important complement to the more traditional process research designs.

Questions have been shown to be a primary vehicle for getting therapeutic work done (Ferrara, 1994; Peräkylä, 1995; Vehviläinen et al., 2008). Questions are grammatically designed in various ways but most appear in a *polar* or *Q-word* format (Stivers, 2010). Question–answer sequences have been shown to perform various kinds of discursive work, and the important role of questioning in institutional discourse is a burgeoning area of investigation (e.g., Freed and Ehrlich, 2010). During medical consultations, for example, questions have been shown to set the agenda, embody presuppositions, display an epistemic position, and make visible response preferences (Heritage, 2010). By setting the agenda, questions make a certain topic of enquiry relevant. Thus, questions may be seen as *topicalizing* a certain problem, relationship issue, past event, and so on; questions may also, in addition to requesting new information, presuppose certain information/knowledge that an answer may implicitly ratify. Because they seek information, questions may imply that therapists have less knowledge or are in an epistemically downgraded position vis-à-vis spouses. Therapists may specifically flag this downgraded knowledge or may even upgrade their knowledge status through specific turn design features; finally, questions invite certain kinds of *response preferences* (Schegloff, 2007), such as *confirmation* following a yes/no question or an answer supplying the requested information following a *wh*-question. Complying with these preferences is considered to be affiliative (Stivers et al., 2011). CA-based studies have begun to identify some important functions of questioning in health assessment and therapeutic interactions, which include the following: attributing the client with positive attributes or so-called optimistic questions (MacMartin, 2008); hypothetical questions to test patients' views or commitments regarding treatment (Speer, 2012); reflexive questions that “*elicit, clarify, and unpack clients” reasoning—their explanations of and reflections on their own experience*” (Gaete et al., 2018, p. 125) and circular questioning to elicit a client's perspective about a co-present other's beliefs or feelings (Peräkylä, 1995; Rossen et al., 2020; Lester et al., 2022).

Our particular interest in this study was to examine the interactive organization of OPQs in couples' therapy. From a general interactional perspective, OPQs invite clients to reflect on the experience (e.g., perspective or feelings) of someone else belonging to their social network (e.g., spouse, friend, and parent). According to Tomm (1987b, p. 5), OPQs “are oriented toward enhancing the ability of family members to distinguish behaviors, events, or patterns that they have not yet distinguished or to see the significance of certain behaviors and events by recognizing their role as links or connections in ongoing interaction patterns”. Furthermore, although there is an element of “mind-reading” associated with these question types, the focus is *interpersonal* and can be used to draw attention to recursive relationship patterns (Tomm, 1987b). Interactionally, OPQs have been shown to elicit “relationship-relevant” talk, draw attention to the systemic nature of problems and overcome resistance (Peräkylä, 1995). Although OPQs have been studied in AIDS counseling (e.g., Peräkylä, 1995), how such questions are used in couples therapy sessions to foster connectedness between spouses remains to be explored.

## 2. Methods

### 2.1. Data and participants

The excerpts analyzed in this project were selected from a prior study designed to examine change processes common to various forms of couples therapy (Horvath et al., 2010).^3^ Clients were offered free time-limited (six sessions) treatment by qualified and experienced couples' therapists with a minimum of a Masters' degree in a relevant field. Therapists provided treatment as they would to their private clients and were paid for their services.

In this study, we aimed to show how CA may be applied to clinically relevant episodes of CT conversation and how OPQs may play a unique role in getting this therapeutic project underway. To illustrate CA in practice, we will be drawing on transcribed extracts of videotaped recordings from couples who have undergone treatment using a combination of narrative and solution-focused techniques (White and Epston, 1990). The excerpts feature the same therapist working with two different couples: Case 10 self-identified “communication” as a common concern^4^, and Case 16 dealt with a legacy of an extramarital relationship; for both cases, the aggregated post-treatment outcome was in the “improved” category.

### 2.2. Analytic approach

CA is the principal method used in this study (Heritage, 1984; Sacks, 1995; Schegloff, 2007). Following Sidnell (2013), our analytic method consisted of three parts: (1) observation; (2) identifying and collecting a corpus; and (3) describing a practice. For the first step, we observed, from watching videos in which the narrative/solution-focused therapist in question worked with couples, that he would commonly ask questions seeking the respondent's perspective of his/her spouse—which we have labeled above as OPQs. We thus wanted to explore this action further. This led us to the second step of identifying and collecting sequences targeting *OPQs* from the transcripts of all 3 cases (18 sessions). Some examples of this question type were as follows: “Where does he get the idea from that you have already made up your mind?”; “Does she know what's going on for you when this happens?”; “Is your going there a problem for her?”; “How does he let you know that your opinion doesn't count?” All sessions were transcribed in this step following the CA transcription conventions (Hepburn and Bolden, 2013). The transcription conventions used in this study are shown in Table 1. For the third step, we drew from Sidnell (2013, p. 83) distinction between *practices of speaking and the actions they implement*. Although the central action is implemented via a question that seeks information, the “practices of speaking” refer to features of turn design that are consequential for bringing about the action of questioning. Thus, we were interested in identifying different ways in which OPQs are grammatically and interactionally designed and how these design features may impact on how the spouses orient to the question. To properly contextualize our analysis of OPQs, we examined these actions in terms of how they were embedded in a sequence of talk (Schegloff, 2007). The minimal unit to examine these constructions involved three parts: Question(Therapist)—Response(Client)—Return Response(Therapist). Responses within the sequence were also considered with regard to two distinct concepts: *Affiliation*, in terms of, for example, whether spouses answered the question straightforwardly (or delayed answering through other-initiated repair, Schegloff et al., 1977) or whether therapists confirmed or disconfirmed the spouse's response—an elaborate discussion on affiliation can be found in Lindström and Sorjonen (2013); *Epistemics*, in terms of how the therapist displayed himself as having less or “downgraded” knowledge or spouses displaying themselves as having “upgraded” knowledge or as lacking the knowledge to answer appropriately (see Heritage, 2012). Other relevant concepts will be briefly explained as they are introduced during the analysis of the extracts. Finally, because our aim was also to explicate how OPQ sequences may be seen as performing interactional work that aligns with systemic therapeutic aims, we also considered how these question sequences accomplished a certain quality of connectedness between the spouses or, more plainly, did relationship work.

**Table 1:** Transcription notation.

**Transcription notation**			
**Symbol**	**Meaning**	**Symbol**	**Meaning**
[	Starting point of overlapping talk	↓word	Markedly downward shift in pitch
]	Endpoint of overlapping talk	↑word	Markedly upward shift in pitch
(1.5)	Silence measured in seconds	.hhh	Audible inhalation, # of h's indicate length
(.)	Silence < 0.2-s
.	Falling intonation at end of utterance	hhh	Audible exhalation, # of h's indicate length
,	Continuing intonation at end of utterance	heh/huh/hah/hih	Laugh particles
?	Rising intonation at end of utterance	wo(h)rd	Laugh particle/outbreath inserted within a word
(word)	Transcriber's guess		
()	Inaudible section	hx	Sigh
wor-	Truncated, cut-off speech	~word~	Tremulous/wobbly voice through text
wo:rd	Prolongation of sound	.snih	Sniff
word=word	Latching (no audible break between words)	huhh.hhihHuyuh	Sobbing
< word>	Stretch of talk slower, drawn out	>hhuh <	Sobbing—if sharply inhaled or exhaled
>word <	Stretch of talk rushed, compressed	((cough))	Audible non-speech sounds
°word°	Stretch of talk spoken quietly	*italics (blue)*	Non-verbal behavior (actor indicated by initial)
word	Emphasis		
WORD	Markedly loud		

## 3. Results: questions seeking other perspectives

A total of 78 examples of OPQ question sequences targeting other perspectives were identified in the data. From this corpus, we identified four distinct practices in which these questions performed interpersonal work: *Soliciting possible optimistic scenarios* (Hypothetical questions; *n* = 9), *drawing attention to other's relationship-fostering conduct* (*n* = 37), *facilitating awareness of other's knowledge* (*n* = 10), and *exploring barriers to productive ways of relating* (*n* = 22). In the first type, *hypothetical questions*, therapists would solicit a spouse's view on how the relationship could become better if some aspect of the other spouse's experience were to change (i.e., knowledge and feelings). “If/then” and “What if…” are common linguistic practices for getting this action underway. For the second category, *drawing attention to other's relationship-fostering conduct*, the therapist would get the spouse to focus on the other spouse's positive contributions to support the relationship in the *here and now*, rather than hypothetically. Commonly turn designs identified were “How does s/he show you Y?” or “Have you noticed her/him doing Y?”. The third category, *facilitating awareness of other's knowledge*, draws unique attention to what the other spouse might not be aware of (or even the spouse's lack of awareness of what the other knows…) and, furthermore, that helping the other spouse to gain this knowledge may produce relationship benefits. Common generic turn designs for this category were “What does s/he know/not know?” Finally, *exploring barriers to productive ways of relating*, this OPQ type gets the spouse to reflect on the reasons why the other spouse might be holding back with regard to conduct or emotions. Some common formats for accomplishing this action were “Why does s/he do/not do/stop doing Y?” Brief conversational extracts illustrating each of these actions are shown in the following subsections.

### 3.1. “If X, would…?”; “What if…?”: soliciting possible optimistic scenarios/hypothetical questions

Hypothetical questions have been described as commonly having an “If/then” or “What if” action format (Peräkylä, 1995; Speer, 2012). A hypothetical question is shown in Extract 1, occurring approximately 15 min into session 2. Prior to this extract, the couple, Melvin and Leyla, had been discussing the importance of their *own* personal relationships with family members (i.e., brother, sister, parent, and cousin). Melvin had mentioned how he valued conversations with his uncle, especially after the death of his father, and the importance he placed on his annual family trips (without Leyla) in order to connect with his family members. Melvin also complained about what he perceived as Leyla not respecting or understanding his need to be with his family.

**Extract 1:**
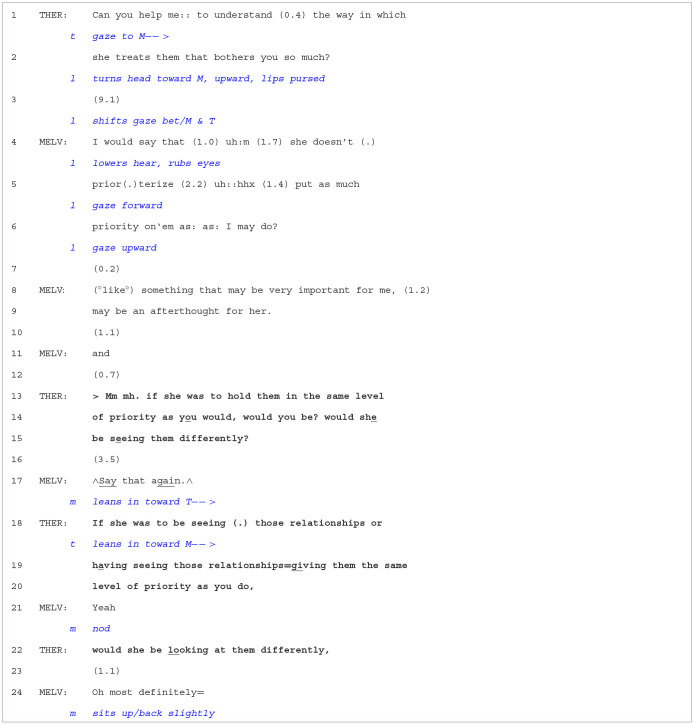
Case 10-2/603.

At the beginning of Extract 1, the therapist asks a question in yes/no interrogative form (Can you…). The question is also designed from what may be termed a “not-knowing position” (Anderson et al., 1992), which clearly positions the therapist as having *downgraded epistemic access* to Melvin's perspective (Heritage, 2012). Thus, by requesting that Melvin “help me:: to understand”, the therapist is seeking more than a “yes” or “no” but rather a clarification or extended telling from Melvin. In lines 04–09, Melvin then produces an account, in which he claims that Leyla does not share the same priorities and may not always recognize what could be important for Melvin. We note that Melvin designs the beginning of his turn as an *opinion* (I would say that), which not only indexes that what he is about to say may be controversial (i.e., Leyla might disagree or have a different viewpoint) but also seems to orient to Leyla's greater epistemic rights. Melvin is, after all, accounting for what Leyla thinks. In response, the therapist asks a hypothetical question that gets Melvin to consider how Leyla might have a different perspective on Melvin's situation if she understood how important certainly family matters were to Melvin. On the one hand, the question proposes an alternative scenario for the couple, one in which Leyla's thinking was more in line with Melvin's. On the other hand, the question generates agreement from Melvin concerning a more productive and positive relationship scenario: Leyla understands Melvin's needs. In line 17, Melvin initiates an *other-repair sequence* with “∧Say that again.∧” (Schegloff et al., 1977) but also leans in toward the therapist, displaying more bodily engagement with what is being proposed. After the therapist's repair, Melvin produces weak confirmation (line 21), leading the therapist to re-do the latter part of the hypothetical question. This, in turn, yields strong agreement from Melvin. To conclude, it would seem that the hypothetical question, by introducing a more productive scenario from Melvin's (but also the couples') perspective, suggested a way out of the dilemma, secured agreement from the client, and gave them a possibility to move forward. Furthermore, this positive movement unfolded sequentially over a series of turns, with the therapist first adopting a “not-knowing” stance concerning Leyla's reasons, which resulted in more elaboration from Melvin. This then created a good context for a hypothetical question that targeted a possible scenario in which Leyla would be more supportive and understanding of Melvin.

### 3.2. “How does s/he show you Y?”: drawing attention to other's relationship-fostering conduct

Some questions targeting other perspectives from a spouse are framed so as to draw attention to what the other spouse may be thinking, feeling, or doing. These questions may appear as getting the spouse to explain or illustrate the other's actions or thoughts/feelings and how these may have a positive benefit for the couples' relationship. They may also be designed in a more tentative manner as what the spouse has *noticed* about the other. An example of this question type is shown in Extract 2, taken from a different couple—Colin and Anna—occurring approximately 40 min into session 2. One of the main issues besetting this couple was that Anna had an affair some years back and Colin recently found out, many years after it had happened. This event caused much tension in the relationship, with Colin feeling hurt and cynical and often expressing these sentiments.

**Extract 2:**
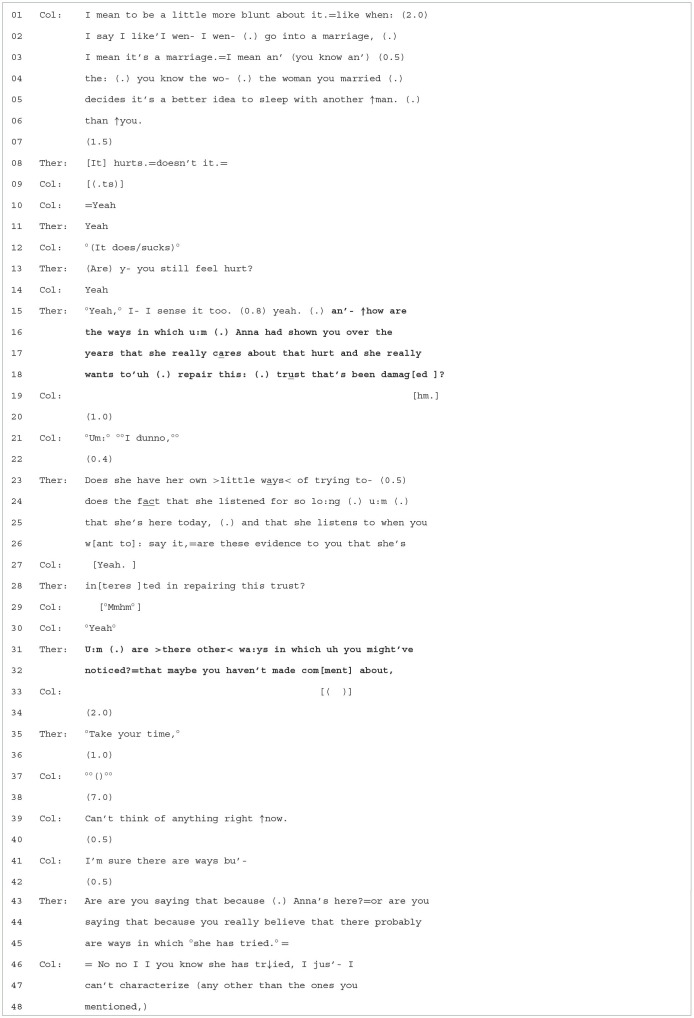
Case 16-2/1366.

Colin begins this extract by recounting the disappointment he felt when Anna slept with another man. In line 08, T affiliates with Colin's turn by *formulating* his feelings of hurt (It hurts.=doesn't it.)^5^, which received immediate confirmation and subsequent upgraded confirmation produced in a quiet voice [°(It does/sucks)°]. In line 13, the therapist picks up on Colin's incipient emotional display by asking whether he *still* feels the hurt, thus highlighting Colin's pain in the present moment of therapy for Anna to witness. Then, following minimal client confirmation, he launches into his OPQ (lines 15–18). The question does various kinds of interactional work. First, it shifts the topic from “Anna hurts Colin” to the more emotionally supportive “how does Anna now show you that she cares/wants to repair the trust”; second, it tries to get Colin to consider more prosocial motives on Anna's part (i.e., she wants to make amends). Although this move could be seen as turning Colin's attention to Anna's positive force in the relationship, rather than focusing on how she has hurt Colin, it may be that the therapist worked to shift the topic too quickly. There is a 1-s pause in line 20, and Colin evinces difficulty in answering by claiming a lack of knowledge (°Um:° °°I dunno,°°) and by speaking even more softly. Moreover, rather than orient to Colin's displayed emotion, the therapist continues to focus on Anna's possible prosocial motives in lines 23–28 (i.e., she's been listening, she's present in couples therapy, wanting to repair the relationship). After receiving minimal confirmation from Colin, he then uses another OPQ to get Colin to name additional prosocial motives Anna might have, independently from what the therapist had already proposed. It also speaks to Colin's hurt without actually directly confronting Anna with this, allowing Anna to “see” Colin's hurt without her being requested to take a position—and maybe become defensive.

The therapist's repeated attempts at topicalizing Anna's positive intentions and getting Colin to contemplate this perspective may, however, have been done too quickly and may not have appropriately attended to Colin's mounting distress at having to confront this painful episode, that is, he may not yet be ready to consider Anna's actions from an alternative, non-hurtful perspective. This is shown in the 2-s pause in line 34 and from the therapist's subsequent “°Take your time,°”. These responses, known as *take-your-times* or TYTs, have been shown to occur in other institutional settings such as caller helplines and police interviews (Hepburn and Potter, 2007; Antaki et al., 2015). They have been shown to manage disruptions in talk rather than affiliate with and elicit more “emotion talk”, that is, TYTs tend to treat emotion as potentially disruptive to the task at hand, which in our extract would be getting Colin to consider Anna's perspective from a prosocial angle. Colin displays great difficulty in engaging with the task, noted by the numerous lengthy pauses and unintelligible speech. He also orients to the expectation that he engages with the therapist's project by providing an account for his not being able to answer (Can't think of anything right ↑now.) but also by asserting that there must be ways in which she demonstrates her caring and trust.

As this extract unfolded, Colin displayed mounting distress and tearfulness on numerous occasions. One option from the therapist could have been to affiliate with his distress by formulating and/or engaging with the distress to some appropriate degree, inviting Colin to continue exploring his hurt (Muntigl, 2020; Muntigl et al., 2023). Instead, the therapist persistently (and quickly) focused his interventions on getting Colin to consider Anna's perspective from a “relationship-building” perspective. If the therapist had invested more affiliation or empathy in his response, before moving on with his *therapeutic project* of getting Colin to recognize Anna's prosocial motives^6^, this may have allowed Colin to deal with his hurt in the moment and might have given him the support and space to collaborate with the therapist. In doing so, however, the conversation would have focused primarily on Colin's emotions and needs, rather than the relationship and systemic implications of his hurt and, most importantly, how Anna could help with his hurt (rather than the therapist). In line 43, the therapist again refrains from engaging with the hurt by instead implying that Colin is avoiding the issue (Are are you saying that because (.) Anna's here?). This leads Colin to become defensive and deny avoidance and instead claim knowledge of Anna's intentions, while not being able to name them.

To conclude, by choosing to stay with the optimistic/positive thread, the therapist consistently supports the foreword movement in the narrative, going from empathizing and validating the “hurt” to noting the possibility that Anna may be participating in the dynamic of emotion by trying to help repair the relationship. Thus, Colin's pain is recast as not just a “private affair” for him to resolve but an interpersonal one in which Anna is an important participant. It also creates a shift from the past to the present, allowing Anna to witness Colin's engagement with his pain.

### 3.3. “What does s/he know/not know?”: facilitating awareness of other's knowledge

OPQs may also solicit a spouse's knowledge about other's knowledge, and these questions often appear in the following turn formats: “What does s/he (not) know about X” and “Does s/he know about X?”, where X generally references other's feelings or important relationship matters.^7^ The implication here is that the other (spouse) might not be aware of the spouse's needs, and, therefore, it may be important to tell him/her. Epistemically, these questions imply that the spouse may be able to take up certain epistemic entitlements by inferring the extent of the other spouse's knowledge in certain relationship events. An example of an OPQ targeting other's knowledge is shown in Extract 3, which is a continuation of Extract 1 with Melvin and Leyla. Recall that the conversation revolved around Melvin's complaint that Leyla does not respect his need to be with his family.

**Extract 3:**
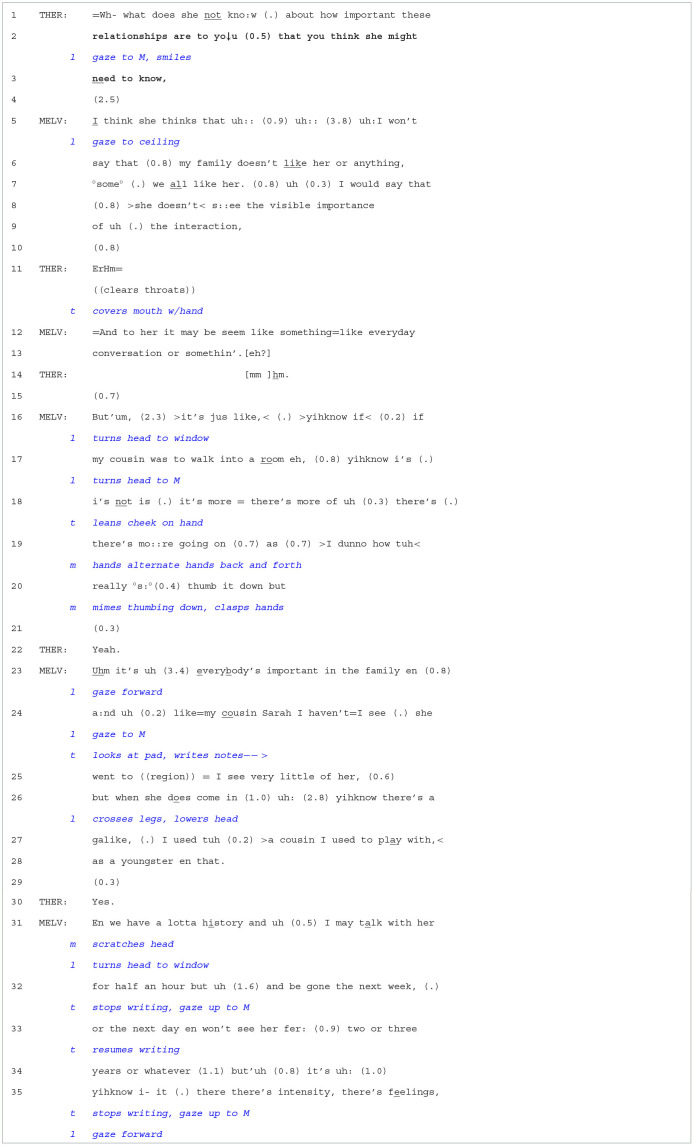
Case 10-2/603.

The therapist continues the prior conversation (from Extract 1) by producing an OPQ (lines 1–3). Epistemically, the first part of the question places Melvin in an upgraded *knowledge* role, ([K^+^]; Heritage, 2012) in which he may infer or gain access to what Leyla does not know about. The second part of the question then solicits what Melvin thinks “she might need to know”. Melvin's response, from line 05 onwards, orients to both these parts of the question. Melvin first reflects on Leyla's knowledge in a downgraded manner (I think she thinks that uh::) and then proposes what Leyla is unaware of (>she doesn't<s::ee the visible importance of uh (.) the interaction,). From line 16, Melvin then goes into significant detail to explain how the interaction between him and his family members is important for Leyla to understand. He first mentions his cousin, recounting that “there's mo::re going on” when he enters the room relationship-wise than is perhaps perceptible to others. He provides another example with his cousin Sarah (line 24). He claims that they have “a lotta history” and that even though they might not see each other frequently or for longer durations of time, “there's intensity, there's feelings,”. To sum up, the therapist's OPQ led Melvin to elaborate not only on his view of Leyla's perspective concerning her lack of knowledge about Melvin's relationship with his family but also occasioned a detailed account of how his relationships with his cousins are very important. Thus, by targeting Leyla's knowledge, the OPQ allowed Leyla to witness Marvin's beliefs about what she is not aware of but also what is important to him and *how* his family relations are deeply meaningful.

OPQs that target other spouses' knowledge may also work to get a spouse to reflect on what s/he believes that s/he knows about other's knowledge. These questions tend to be used in a context in which the spouse does not yet realize that his or her spouse already possesses the requisite knowledge. An example is shown in Extract 4, which is a continuation of Extract 3, involving Colin and Anna.

**Extract 4:**
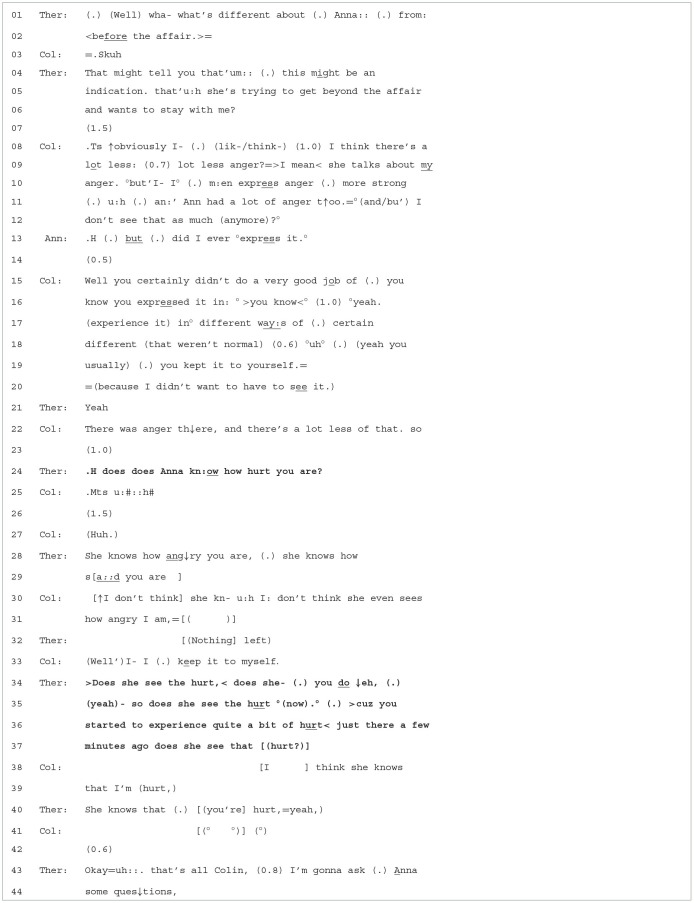
Case 16-2/1366.

As can be recalled from Extract 2, the conversation revolved around Colin's feelings of hurt and the contrasting therapeutic agenda of getting him to consider Anna's prosocial motives of building trust and caring about his hurt. The therapist continues with his agenda by asking Colin to consider the positive, caring ways in which Anna has been behaving following the affair (lines 01–06). The question contains a premise that Anna has changed, then introduces in a downgraded manner the possibility that she is “trying to get beyond the affair”, and offers an optimistic assessment that she wants to stay. In line 06, the use of “me” seems to offer a “first person” option to Colin. Colin, however, is still hearable upset, as evidenced by his *snorty sniff* in line 03 (Hepburn and Potter, 2007). Colin's response, while addressing different positive ways that Anna has changed, appears more disaffiliative than affiliative. For example, although he concedes that Anna has a lot less anger, he prefaces this assertion with “↑obviously”, which may have two implications. One is that what he is about to say is not particularly newsworthy, and the second is that he is challenging the relevance of the question or even the presupposition of its askability (Stivers, 2011).^8^ Colin then goes on to further challenge Anna's changed, positive behavior by noting that “she talks about my anger”, implying that Anna still may have anger issues, which he then states explicitly (an:' Ann had a lot of anger t↑oo.). He does, however, concede that her anger has subsided.

What then follows from Colin's response is a disagreement sequence. In line 13, Anna denies the prior claim that she was often angry. Colin, in turn, responds with a disagreement that, on the one hand, claims that she did express anger and, on the other, she would keep her anger to herself because she did not want Colin to witness it. Colin's position is stated more succinctly in line 22 (There was anger th↓ere, and there's a lot less of that. so). Colin is also displaying *low intensity upset* during his turn (Muntigl et al., 2023), as shown by his quiet voice and frequent pausing and this display may be occasioning the therapist's next move in line 24, which addresses Colin's hurt in an OPQ form: does Anna kn:ow how hurt you are?'. Thus, the topic is shifted away from a disagreement to Colin's feelings in the present moment—note that the therapist uses present tense “are” rather than past tense “were”. The question also positions Anna as someone who is able to access Colin's emotive state.

Colin, however, appears choked up and has difficulty responding (lines 25–27), which leads the therapist to assert Anna's knowledge of his anger and sadness. In lines 30–31, however, Colin counters the therapist's assertion by stating that she is not aware of his anger and accounts for this with “I- I (.) keep it to myself”. Thus, Anna cannot access Colin's feelings because he does not reveal them to her. Thereafter, in lines 34–37, the therapist produces another OPQ that again targets what Colin thinks Leyla knows: >Does she see the hurt, <. In doing so, the therapist again shifts the topic, but this time from Colin's “anger” to his “hurt”. After receiving visual confirmation from Leyla that she does (you do ↓eh, (.)), he takes this as a “go ahead” to focus on Colin's hurt emotions in the *here and now*, which implies that Colin's hurt is not a “private affair” but something that has a legitimate place in the present conversational moment. This focus on the present moment is linguistically accomplished throughout the rest of the therapist's turn with the following expressions: “now”, “you started to experience” and “just a few minutes ago”. Colin confirms the therapist's question, which is followed by the therapist's return confirmation, thus producing an *empathic moment* (Heritage, 2011; Muntigl, 2020), in which mutual understanding surrounding Leyla's knowledge of Colin's hurt is achieved.

To conclude, we argue that the OPQs work to break a relationship pattern in which Colin suffers privately” and Anna feels guilty for Colin's guilt but also privately.^9^ The therapist's OPQs guided Colin into having to confront Anna's knowledge of his suffering, forcing him to acknowledge it, with the aim of being able to move past it at some later point. Through these questions, the therapist is attempting to generate a new way of relating to each other by topicalizing Colin's hurt in the present moment and by getting it out in the open for it to be noticed, acknowledged, and explored by both spouses.

### 3.4. “Why does s/he do/not do/stop doing Y?”: exploring barriers to productive ways of relating

OPQs may also be used to explore the reasons and motives of other's conduct. Questions such as “why do you think he does that?” or “why does he stop doing it?” are typical examples of such questions. Extract 5 provides an example of an OPQ that targets other's motives. This extract from session 2 involves Melvin and Leyla and occurs only a few minutes after Extract 3. Recall that both Melvin and Leyla find family to be extremely important and that Melvin has complained that Leyla treats his need to be with his family with contempt. The therapist asks a question to explain Leyla's reasons for being contemptuous and explain this contradiction, which prompts Melvin to tell a story about how Leyla does not respect his need to be with his family.

**Extract 5:**
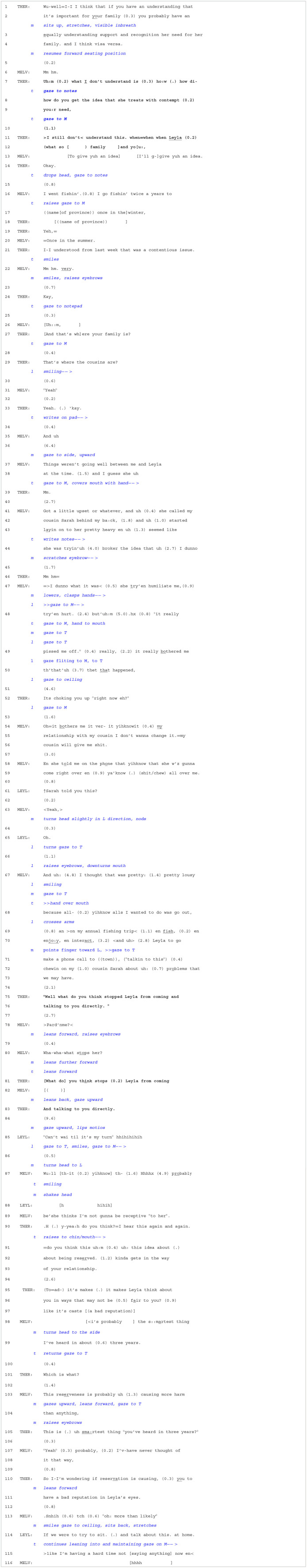
Case 10-2/815.

At the beginning of the Extract, the therapist directs a question to Melvin, asking him to explain how Leyla does not respect Melvin's needs (she treats with contempt (0.2) you:r need,). The therapist adopts an epistemic frame (what I don't understand) that not only makes explicit his downgraded knowledge about this issue but also signals Melvin's greater knowledge and entitlement to speak about Leyla's possible reasons for disrespecting his needs and that his knowledge about her motives is relevant here. Melvin then takes up this opportunity in line 13 by beginning with a *story preface* (Sacks, 1995) that signals an upcoming story that will respond to T's question (I'll g-give yuh an idea.) and after, by launching into a storytelling episode about his fishing trip. During Melvin's story, in line 16, T takes up a turn that does important discursive work. It underscores that this is not a first-time telling (i.e., Melvin had mentioned that he frequently visits his relatives to go on a fishing trip in the last therapy session) and makes salient the affectual nature of this issue (i.e., its *contentious*).

From line 35 onwards, Melvin begins to tell the main narrative. After mentioning that his relationship with Leyla was not going well, he lists the different moral transgressions committed by Leyla, all of which centered around Leyla's phone call to Melvin's cousin Sarah. For instance, Melvin uses many negative expressions that index various forms of misconduct such as “behind my ba:ck.”, “laying on to her pretty heavy”; “broker the idea”. Following a *continuer* from T in line 46 that encourages Melvin to continue with his story (Muntigl and Zabala, 2008), he first suggests that her misconduct may have been purposeful (“try'en humiliate me, (0.9) try'en hurt.”) and then states how Leyla's actions had affected him emotionally (°it really pissed me off.°; it really (2.2) really bothered me). It should also be noted that, in lines 48–51, the delivery of Melvin's talk also changes in terms of the length and frequency of his pauses but also, more importantly, his softened and quieter voice. In line 52, the therapist seems to pick up on this change by producing a noticing that calls attention to Melvin's displayed emotion in the here and now (its choking you up °right now eh?°), thus moving the focus toward how this event that happened in the past is having a significant impact on him in the present moment of therapy. With this move, the therapist is able to confirm, and convey empathy with, Melvin's feelings of distress (Muntigl et al., 2014; Ford and Hepburn, 2021).

Research has shown that noticings tend to implicate a subsequent affiliative response from the client such as confirmation or even more elaborate “feelings talk” (Muntigl and Horvath, 2014). We see in line 54 that Melvin strongly confirms the noticing (Oh=it bothers me) but then proceeds to discuss how Leyla's phone call could have created a rift in the relationship between him and his cousin Sarah (my relationship with my cousin I don't wanna change it.). This is because his cousin had planned to visit Melvin and berate him for his actions (i.e., chew all over him). Thus, Leyla's phone call is portrayed as potentially creating multiple lines of disaffiliation, not only between him and his wife but also between him and his cousin as well. This may explain Leyla's response in line 61, in which she expresses surprise at Sarah's action (↑Sarah told you this?) and, therefore, that she did not know about Sarah's plans. By implying that it was not her intention to get Sarah to berate Melvin, Leyla displays an attempt to repair (at least part of) the mounting disaffiliation between her and Melvin. By way of response, however, Melvin continues to criticize Leyla's actions as “lousy” and to assert his own innocence in the matter (“alls I wanted to do was go out, (0.8) an on my annual fishing trip. …”).

Faced with this complaint story in which Melvin provides a detailed account of Leyla's misconduct and his own innocence, the therapist could respond with an affiliative and empathic move—such as a formulation—that validates Melvin's emotional experience. The risk in doing so, however, would be to deepen and support Melvin's portrayal of Leyla as the wrongdoer and as Melvin as the victim. Instead, the therapist poses a question to Melvin that does not affiliate with Melvin's complaint but rather provides an alternative perspective and explanation of the story events. T's question—“**°**what do you think stopped Leyla from coming and talking to you directly**°**.”—performs a wide range of important therapeutic work: It presupposes that Leyla may have tried or wanted to talk to Melvin directly about the trip (and what is bothering her), it presupposes that something had “stopped” her from doing that, and, finally, it frames these presuppositions as a question in which Melvin may himself provide the answer. The OPQ, therefore, marks an abrupt shift in the way in which the events told by Melvin have thus far been constructed and conceptualized. In line 78, Melvin seems to orient to this “radical shift” by expressing a lack of understanding or incredulity by producing what in CA parlance is termed an *other-initiated repair* (Schegloff et al., 1977) and by leaning in toward T during which his eyes widen. Following a brief silence, Melvin re-initiates repair (“wha- wha- what stops her?”) and T, in lines 81–83, offers a repair by repeating his question but this time by incorporating Melvin's present tense use of “stops”. In this subtle move, the focus of attention moves from what may have stopped Leyla in that specific situation to what may generally stop her from talking to Melvin both in the past and present. What then follows is a long pause in which Melvin makes verbal signs of contemplation (tapping his foot, moving “mouth movements” with closed lips). After Leyla makes a remark in line 85 that jokingly suggests that she should take up the turn and answer, Melvin provides a response that is prefaced by some hesitation and possibly some degree of distress (note the long exhalation and long pauses). But now rather than continuing to complain about Leyla, he offers up his own behavior as an obstacle to communication between them (“be' she thinks I'm not gonna be receptive to her.”). Thus, with this seemingly subtle questioning move, the therapist is able to extend Melvin's complaint about Leyla as the principal wrongdoer toward a more reflective mode in which both persons' actions are made accountable for co-constructing a certain relationship pattern.

Melvin's reservation continues to be a topic in the remainder of this Extract. In lines 90–97, the therapist seeks confirmation from Melvin about the negative impact of reservation (kindagetsinthatwayofyourrelationship;itmakesLeylathink aboutyouinwaysthatmaynotbe(0.5)fairtoyou.). Melvin's next responses are not only strongly confirmatory, but they also underscore the magnitude in which reservation may be acting as a negative force in the relationship. First, the relevance of “being reserved” is treated as highly significant (its probably the s::martest thing I've heard in about (0.6) three years.); second, reservation is characterized as a new perspective on the relationship or a “leap forward” in thinking (“I'v-have never thought of it that way.”); and third, they convey strong affiliation with the therapist's view that reservation could be a relationship problem. Melvin's responses, therefore, seem to index alliance building between him and the therapist both in terms of strengthening the therapeutic relationship and also in terms of being mutually aligned in the joint task of how productive couples therapy may now proceed.

## 4. Discussion

Our goal in this study was 2-fold: to explore some of the different ways in which OPQs can be used to generate forward movement in CT and to explicate some of the important relational functions of OPQs and how these are realized in sequence. Our CA focus on the turn design features of these questions revealed that different linguistic practices may be employed to implement the action of *other-perspective* questioning in unique ways. Thus, our study extends past studies, for example (Peräkylä, 1995), by identifying three additional ranges of practices—beyond hypothetical questions targeting other perspectives—through which this question type may be implemented. Our investigation of the four specific types of OPQs also allowed us to explore how some important “systemic”, interpersonal work was set in motion: soliciting optimism in the relationship, fostering the couples' healthy relationship, promoting awareness of the interlinked aspects of the couple's experiences and challenges, and promoting of novel and creative ways of contextualizing their relational dynamics.

Our CA analyses allowed us to highlight how these OPQs are produced in sequence, yielding a more positive conceptualization of the “others” included or implied in the question (e.g., Extract 1, lines 13–15; Extract 2, lines 15–18 and 31–32). In other examples, we were able to identify how, using OPQ formats, the therapist topicalized the notion that each member of the couple had some private ideas about the other's thoughts and, by raising this as a topic for discussion, created room for re-negotiating these private beliefs and highlighted the important interconnection between these beliefs and their relationship (e.g., Extract 4, lines 24; Extract 5 lines 7–9). In other cases, OPQs were crafted in a way that offered the clients new conversational resources with the potential to re-engage with their partner in different terms, e.g., Extract 5 lines 90–104. In this last example, we saw evidence of an enthusiastic “uptake” of the resources offered (lines 98–99). Thus, through CA, we were able to highlight how OPQs were not delivered “pre-baked” but built sequentially, interactively, and responsively on a turn-by-turn basis, sometimes repeated and reinforced.

OPQs often included an offer displaying one's knowledge to the client and, consistent with Peräkylä (1995) findings for AIDS counseling, downgraded the therapist's epistemic access. Thus, the spouse was placed in an upgraded epistemic position to offer knowledge of the *partner's* knowledge, feelings of motives, which were often followed by a positive hypothesis of the partner's intent. For example, in Extract 5 (lines 75–76): “°what do you think stopped Leyla from coming and talking to you directly°” Crafted this way, the preferred response to a “wh” question would address the part “what stopped” but also invite Melvin to take a position on the relationally positive/optimistic proposition that follows: “coming to you directly”.

As Peräkylä and Vehviläinen (2003) study on “professional stocks of interactional knowledge” has argued, CA is well suited to investigate virtually any theoretical approach within the helping professions, with respect to the realization of the theoretical models in actual practice. This study gives credible evidence for the possible pivotal role of OPQs in launching conversational sequences working to achieve therapeutic ends, as accentuated in systemic-narrative therapies. Although the broader therapeutic functions identified in this study may also have been initiated by other kinds of conversational actions or sequences, we argue that OPQs seem to be especially attuned to getting interpersonal work between the spouses underway, work that gets spouses to consider spousal actions from an alternative, often more prosocial, perspective. Our excerpts were drawn selectively from treatments with a systemic-narrative focus. This choice provided us with a variety of clear and interesting examples of the use of questions in therapy and afforded the opportunity to illustrate how examining questions qualitatively through the lens of CA can complement quantitative studies. While examples we drew on were thus constrained, we believe that our research may be a useful “first step” in generating knowledge of how questions are built to perform specific roles in therapy. Furthermore, we anticipate that studies like the one we present will be useful for training therapists in understanding how to use questions creatively and also to illustrate potential challenges and pitfalls in using this question format in therapy.

## Data availability statement

The original contributions presented in the study are included in the article/supplementary material, further inquiries can be directed to the corresponding author.

## Ethics statement

The studies involving human participants were reviewed and approved by Simon Fraser University Research Ethics Board. The patients/participants provided their written informed consent to participate in this study.

## Author contributions

PM: designed the study, performed data analysis, supervised data analysis, and wrote the article. AH: performed data analysis and wrote the article. All authors contributed to the article and approved the submitted version.

## Footnotes

^1^For an extended theoretical discussion of the forms of questions in therapy, see Hill (2020) and McCarthy et al. (2021).

^2^For exceptions, see Williams (2023) on frequency of use and Hill (2020) and Anvari et al. (2020) on relation to outcome.

^3^Clients signed informed consent forms permitting the use the transcripts for research purposes. The transcripts are anonymized to remove the potential links to the actual persons involved; the research protocol was approved by the University Ethics Review Board.

^4^A third couple, Case 8, was also analyzed for OPQs, but we chose not to include extracts from this couple in this study mainly for reasons of space. We chose instead to focus on two cases in which OPQs were deployed and negotiated over longer stretches of conversation.

^5^By *formulating*, in this position in sequence, we mean ‘providing the upshot' of what Colin had felt when he discovered his wife had slept with another man (Antaki, 2008).

^6^According to Peräkylä (2019, p. 267), a therapeutic project consists of “the overall goal setting and structuring of the interaction that is meant to help this particular client to overcome his/her particular obstacles in his/her behavior, social relations, or internal life.” We think that the question's focus on the spouse's possible prosocial ‘motives' constitutes one such project.

^7^Verbs other than *know* are also used, such as *understand* or *recognize*.

^8^Stivers' work examined responses to questions containing “of course”, which seems similar to “obviously”.

^9^By relationship pattern, we mean that each client may be assuming that their feelings were “private”, not accessible to the other and experienced “within the self”. The therapist's questions may be fostering a new systemic concept; that emotions arise from a relational context where each has an important stake in gaining access to the other person's own feelings as well as the assumptions about the other. Treating emotions as *inter*subjective (versus intra-subjective) has the potential of opening up space to re-consider “where the other is coming from” and thus a more flexible negotiation in the relationship.
